# Classifying Coding DNA with Nucleotide Statistics

**DOI:** 10.4137/bbi.s3030

**Published:** 2009-10-28

**Authors:** Nicolas Carels, Diego Frías

**Affiliations:** 1 Fundação Oswaldo Cruz (FIOCRUZ), Instituto Oswaldo Cruz (IOC), Laboratório de Genômica Funcional e Bioinformática, Rio de Janeiro, RJ, Brazil; 2 Universidade do Estado da Bahia (UNEB), Departamento de Ciências Exatas e da Terra, Salvador, BA, Brazil. Email: nicolas.carels@gmail.com

**Keywords:** genomics, universal correlation, purines bias, coding features, open reading frame, ancestral codon

## Abstract

In this report, we compared the success rate of classification of coding sequences (CDS) vs. introns by Codon Structure Factor (CSF) and by a method that we called Universal Feature Method (UFM). UFM is based on the scoring of purine bias (Rrr) and stop codon frequency. We show that the success rate of CDS/intron classification by UFM is higher than by CSF. UFM classifies ORFs as coding or non-coding through a score based on (i) the stop codon distribution, (ii) the product of purine probabilities in the three positions of nucleotide triplets, (iii) the product of Cytosine (C), Guanine (G), and Adenine (A) probabilities in the 1st, 2nd, and 3rd positions of triplets, respectively, (iv) the probabilities of G in 1st and 2nd position of triplets and (v) the distance of their GC3 vs. GC2 levels to the regression line of the *universal correlation*. More than 80% of CDSs (true positives) of *Homo sapiens* (>250 bp), *Drosophila melanogaster* (>250 bp) and *Arabidopsis thaliana* (>200 bp) are successfully classified with a false positive rate lower or equal to 5%. The method releases coding sequences in their coding strand and coding frame, which allows their automatic translation into protein sequences with 95% confidence. The method is a natural consequence of the compositional bias of nucleotides in coding sequences.

## Introduction

With the continuously growing of sequencing activities, the demand for automatic gene finding processes remains a priority. The strategies of gene localization relies on extrinsic (homology search) and intrinsic (pattern search) methodologies. High confidence levels can be given to the extrinsic methods even at small sequence size (<300 bp) given the negligible probability of achieving good alignment just by chance. Extrinsic methods definitely allowed the improvement of gene annotation reliability by comparison with well documented protein families. However, they depend on reliable sequences for comparison. In addition, they may fail to recognize protein regions that are not associated to enzymatic domains because of their lower conservation rate.

In eukaryotes, intrinsic gene detection was classically considered in several steps: (i) coding sequences (CDS) detection, (ii) intron detection, (iii) 5’ and 3’ gene extremities search, (iv) promoter localization, and (v) gene structure confirmation by local base composition profiling.^1^

Methods for CDS detection^2^–^4^ were initially based on the codon usage^5^ and were classically solved by Markov chains^6^ and neural network based algorithms. 7–11 Later on, multiple stage probabilistic models called Hidden Markov Models integrating the whole information about gene structure were proposed.^12^,^13^ The success rate of these models depends on the representativeness of training sets.^14^ The concept of a self-training algorithm has been introduced with the purpose of extending the applicability of these models to the gene search in genomes with little or no previous information.^15^,^16^

Methods based on nucleotide statistics were also introduced. Zcurve, one of these methods,^17^–^19^ is used for the whole genome characterization,^20^ but it also requires a training step. Other methods based on nucleotide statistics are sensitive to nucleotide periodicity in CDS. 21–26 These methods are largely independent of the biological species under consideration and, therefore, are not supposed to be trained. They offer the advantage to be tolerant to the codon usage, but suffer lack of sensitivity for the detection of CDSs with sizes below 400 bp. Among these methods AMI (Average Mutual Information^25^) and SRM (Spectral Rotation Measure^26^) are the most relevant.

The success rate of methods based on the detection of the so called ‘ancestral codon’ characterized by the RNY pattern^27^ has been recognized to be higher at sequence sizes below 350 bp^28^ than methods based on nucleotide periodicity in CDS. The implementation by Nikolaou and Almirantis^29^ maximizes a function based on the Codon Structure Factor (CSF) that measures the codon asymmetry in the 3 frames. The maximum of this function tells if the sequence must be considered for coding or not by reference to a given threshold.

Compared to CSF, the method introduced by Carels et al^30^ implements a function based on purine bias (Rrr) and stop codon frequency. For commodity, we decided to use the acronym UFM (for Universal Feature Method) to refer to this method. UFM is largely independent of codon usage and is the first among the methods based on nucleotide statistics that is able to classify the coding frame among the six possible frames of a given coding ORF without any parametric adjustment.

The current tendency in CDS classification is to combine several intrinsic methods and to identify their domain of convergence. This allows the reduction of false positive rate and, as a consequence, to increase their success rate at small sequence size, i.e. <300 bp.^28^ Intrinsic methods can also be combined with extrinsic methods, with the same purpose.^31^

Here, we focus on methods of CDS/intron classification that do not need training steps and in particular on methods based on the detection of the RNY pattern (CSF, UFM) because their success rate is higher than the methods based on the detection of nucleotide periodicity (AMI, SRM). Actually, the success rates of AMI and SRM typically vanish for sequences smaller than 350–400 bp.^32^ We first compare the success rate of CDS/intron classification of CSF with that of a new version of UFM. Secondly, we show how UFM scoring can be improved to classify coding and non-coding CDSs (~250 bp) of *Homo sapiens*, *Drosophila melanogaster* and *Arabidopsis thaliana.* We show that more than 80% true positives of CDSs of *Homo sapiens* (>250 bp), *Drosophila melanogaster* (>250 bp) and *Arabidopsis thaliana* (>200 bp) are successfully classified with a false positive rate lower or equal to 5%. The method releases coding sequences in their coding strand and coding frame, which allows their automatic translation into protein sequences with 95% confidence.

## Materials and Methods

### Sequence materials

We built datasets with coding sequences (CDS) of three model species including *Arabidopsis thaliana* (CDS = 1206, GC3 = 25%–65%), *Drosophila melanogaster* (CDS = 1262, GC3 = 40%–85%) and *Homo sapiens* (CDS = 1199, GC3 = 30%–90%).

We retrieved complete nuclear CDSs from GeneBank (release 137–15 August 2003) using the ACNUC/QUERY retrieval system^33^ with the options: t = cds et no o = plastid et no o = mitochondrion et no k = partial et no k = est. Then, we used the bibliographical references reported under the field MEDLINE in the features to build datasets of experimentally proven genes as follows: (i) the MEDLINE identification numbers were used to retrieve the abstracts of the corresponding genes from the NCBI server (PubMed), through a CGI interface (PERL); (ii) those abstracts were then screened to eliminate mitochondrial and chloroplast genes as well as (retro)transposons and references based on any kind of automatic *in silico* process. To eliminate redundancy from CDS samples, we looked for homology between sequences using BLASTN with the “-e” option equal to 0.0001. A cleaning procedure was then applied to the BLASTN file in order to eliminate the sequences implied in a homologous pair with the highest hit when it was above a given identity level. The identity level above which two sequences were considered redundant was set to 90% over 90% of the homologous regions with the shorter sequence of the pair.

All sequences of our test samples were started with an ATG, and ended with a stop codon and did not have in-frame internal stop codon, which warrant that they were in frame +1. To allow statistical comparison, sample size was normalized to 1000 per species.

We tested the success rate of CDS/intron classification with the CDS samples of *A. thaliana*, *D. melanogaster*, *H. sapiens* just described and samples of intron sequences of these species retrieved from http://hsc.utoledo.edu/bioinfo/eid/index.html.^34^ For purpose of normalization, CDS and intron datasets were built by cutting pieces of fixed size extending from the 5’ side to the desired sequence size. Two sets of intron sequences were prepared according to sequence availability. The first was obtained by selecting introns ≥1000 bp. Datasets of sequence fragments of 300, 400, 500, 600 bp were then prepared from the 5’ side of coding and intron sequences. This sequence material was used to compare the success rate of CDS/intron classification by Codon Structure Factor (CSF) and Universal Feature Method (UFM) (see below). The size of sequence samples of this experiment was normalized to 500 per species. The second was obtained by selecting introns ≥500 bp. Datasets of sequence fragments of 150, 200, 250, 300, 350 and 400 bp were then prepared from the 5’ side of these sequences. This sequence material was used to compare the success rate of the UFM in various experimental conditions (see below). The sample size for this experiment was normalized to 1000 per species.

### Conventions and classification contexts

The translation of a nucleotide fragment by a ribosome occurs in opposite ways on both strands of the corresponding double strand DNA. Therefore, the nucleotide sequence of one strand is the reverse complement of the other. By convention, the coding strand of a CDS is indicated by “+” and the complementary strand by “−”. By extension, the frames on the coding strand are indicated by *k* ∈ {+1, +2, +3} and are in-frame with the 1st, 2nd and 3rd positions of codons, respectively. On the complementary strand, the non-coding frames are indicated by *k* ∈ {−1, −2, −3} and are in-frame with the reverse complement of the 1st, 2nd and 3rd positions of codons, respectively. By default, we considered that sequences were on the “+” strand. Therefore, the corresponding sequences on the “−” strand were obtained by calculating their reverse complement.

We took two classification contexts into account: the first concerned the comparison of CSF and UFM for CDS/intron classification and the second concerned the implementation of UFM in an algorithm that is compatible with *ab initio* gene-finding.

In the first case, CDSs and introns were considered on the whole, to be consistent with former investigations on CSF.^29^ By consequence, CSF and UFM functions were calculated among the three positive frames, only. In the second case, we wanted to measure the influence of the *ab initio* gene-finding context on the performance of UFM. In that context, neither the coding strand nor the coding frame is known *a priori*. This implies the calculation of UFM for ORFs over the six frames. By ORF, we mean a stretch of DNA that starts and ends with stop codons (TAA, TAG, TGA) or sequence extremities separated by a whole number of nucleotide triplets.

### Scoring the coding potential of ORFs with UFM

The methodology used here involves four steps: (i) extraction of all ORFs from all frames (three in CDS/intron classification context and/or six in ab initio classification context) of a given DNA sequence; (ii) elimination of the ORFs without the purine bias characteristic of CDSs; (iv) selection of the largest of these ORFs if and only if its size exceeded the selected threshold size and (v) declaration of the selected ORF as putatively coding.

The scoring of the contribution of purines bias to CDSs was carried out by computing the relative frequencies, *P_i_*_(_*_j_*_)_, of the four nucleotides *i* (*i*∈ {A,C,G,T}) in the three positions *j* of triplets (*j*∈ {1,2,3}) over all frames. The probabilities *P_i_*_(_*_j_*_)_ were computed as the ratio of a given occurrence to the number of contiguous triplets *N* = *n*/3 where *n* is the nucleotide number in the sequence. The contribution of purines (A and G) was evaluated in the three positions of triplets by computing the product of their relative frequencies *P_A_*_(1)_*P_G_*_(1)_ over all frames. We also computed the number of stop codons (TAA, TAG, TGA—that we denoted STOP) and the product of the relative frequencies of C, G and A, i.e. *P_C_*_(1)_*P_G_*_(2)_*P_A_*_(3)_, over all frames.

Using the terms just described, we set up a feature for the diagnosis of coding ORFs as follows: *f_k_* *P_A_*_(1)_*P_G_*_(1)_/(*P_C_*_(1)_*P_G_*_(2)_*P_A_*_(3)_ +STOP+W) where (i) STOP is the number of stop codons in-frame with the frame *k* considered, (ii) *k* ∈ {+1,+2,+3} in the CDS/intron classification context or *k* ∈ {−1, −2, −3, +1, +2, +3} in the *ab initio* classification context, as noted above and (iii) W is a constant whose most appropriate value was found to be 0.01.

A sequence was classified as coding when (i) the difference between the maximum (*f_max_*) and the minimum (*f_min_*) values of *f_k_* over all frames *k* was higher or equal to a threshold *τ_UFM_* whose optimization is described below. In the following, we use the acronym *PBI* (for Purine Bias Index) to refer to the quantity *max*(*f_k_*)*–min*(*f_k_*). Therefore, UFM classifies a sequence as coding when *PBI* ≥ *τ_UFM_*.

### Comparing CSF and UFM for CDS/intron classification

We computed the Codon Structure Factor (CSF) by calculating the quantity *CSF_k_* = ∑ *R_I J L_*/(*P_L_*_(1)_*P_J_*_(2)_*P_I_*_(3)_) where (i) *n* is the sequence size; (ii) *R_I J L_* is the frequency of triplets having the nucleotides *I*, *J* and *L* ∈ {A,C,G,T} in 1st, 2nd and 3rd positions, respectively; (iii) *P_L_*_(1)_, *P_J_*_(2)_ and *P_I_*_(3)_ are the frequencies of the nucleotides *L* at the 1st position, *J* at the 2nd position and *I* at the 3rd codon position, respectively. Both *R* and *P* are frequencies relative to *n*. The maximum of the three values *CSF_k_* is taken to be the *CSF*, i.e. *CSF* = *max*(*CSF_k_*).^29^ The sequence is classified as coding if CSF ≥ *τ_CSF_*, where *τ_CSF_* is a threshold whose optimization is described below.

### Optimization of classification thresholds

The optimal threshold values of CDS/intron classification by CSF (*τ_CSF_*) and UFM (*τ_UFM_*) were estimated by fixed-point optimization^35^ of the harmonic mean of sensitivity and specificity. The resulting function is a F-score^36^= 2*Sn*Sp/(*Sn* + *Sp*)where *Sn* =*TP/*(*TP* + *FN*) is the sensitivity and *Sp* + *TN/*(*TN* + *FP*) the specificity, i.e. TP = “true positives” (sequences correctly classified as coding), FP = “false positives” (sequences wrongly classified as coding), TN = “true negatives” (sequences correctly classified as non-coding) and FN = “false negative” (sequences wrongly classified as non-coding).

According to this procedure and the 24 datasets of 500 sequences of this study (datasets of 300, 400, 500 and 600 bp for CDSs and introns of *H. sapiens*, *D. melanogaster* and *A. thaliana*), we calculated that *τ_CSF_* = 75 and *τ_UFM_* = 1.

### Classifying coding and non-coding ORF with UFM

The procedure of coding ORF diagnosis that we describe below (see algorithm) involves (i) the identification of ORFs with the typical purine bias of CDSs (*PBI* ≥ 1), (ii) the extraction of the largest among these putative coding ORFs and (iii) two filtering steps (*a priori* and *a posteriori*) that reduce the rate of false positives. In these filters, we calculated (i) GC as the frequency of G + C relative to the sequence size, (ii) GC2 as the frequency of G + C in 2nd position of triplets relative to the triplet number and (iii) GC3 as the frequency of G + C in 3rd position of triplets relative to the triplet number.

We used two conditions of *a priori* filtering both based on the distance of GC3 vs. GC2 to the orthogonal regression line (GC3 = 7.14*GC2-241.5) of the *universal correlation*,^37^ i.e. “accept ORF as coding if GC2 < (GC3 + 20)/3” (equivalent to “accept ORF as coding if GC3 > 1.5*GC-27”) when “GC > 60%” (filter 1) or when “GC > 50%” (filter 2), i.e. these two filters only differs by their GC cut off. These *a priori* filters allow the separation of putative coding ORFs from random non-coding ORFs (those with GC3≈GC2) in GC-rich sequences (found at least in warm-blooded vertebrates, *Gramineae*, and *Chlamydomonas reinhardtii*, with a GC higher than 60%).

We used one condition of *a posteriori* filtering that is “accept ORF as coding if G1 > G2” (filter 3). This condition is known to be true in >94% of coding frames of complete CDSs^30^ and also allows the filtering out of false positives. However this filter was found to increase the number of false negatives therefore slightly reducing the sensitivity of the method.

### Algorithm for the implementation of UFM

1. Load the sequence into the program,
2. Scan the three frames in the “+” and “−” strands for stop codons,
3. For each the “+” and “−” strands, construct a table with the ORFs of the three frames,
4. For each strand, scan the corresponding ORF table and:
  - measure the ORF size,
  - if the ORF under consideration is larger than a selected size threshold:
    ○ calculate *f* over the six frames of that ORF and return it if *PBI* ≥ 1,
    ○ *a priori* filtering,
5. Chose the largest ORF among “+” and “−” ORFs returned by loop 4,
  - if the two largest ORFs from “+” and “−” strands are of the same size, chose the one with the highest score *f_1_*, i.e. *f_max_* in frame +1,
6. *A posteriori* filtering.

## Results

### Comparison of the performances of UFM and CSF methods

**Figure 1.**
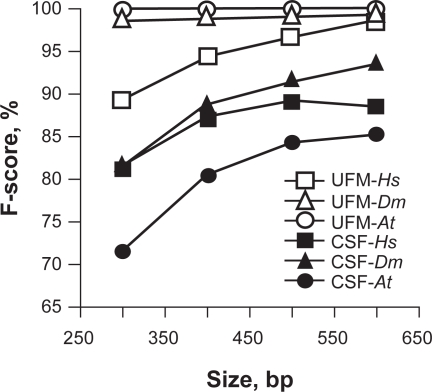
Plot of F-score for CDS/intron classification by CSF (black symbols) and UFM (white symbols) in *H. sapiens* (*Hs*), *D. melanogaster* (*Dm*) and *A. thaliana* (*At*).

The values of the classification thresholds (*τ*) that we found after fixed point optimization of the harmonic mean of sensitivity (Sn) and specificity (Sp) were *τ_CSF_* = 75 and *τ_UFM_* = 1 for CSF and UFM, respectively. Given *τ_CSF_* and *τ_UFM_*, the *F-score* values of CDS/intron classification by UFM were higher than those by CSF in all three species with differences of 8%, 11% and 24%, on average, in *Homo sapiens*, *Drosophila melanogaster* and *Arabidopsis thaliana*, respectively (Table 1, Fig. 1).

The success rate of CDS/intron classification with UFM was found to be higher in *A. thaliana* and *D. melanogaster* than in *H. sapiens* (Fig. 1) suggesting fundamental differences in the intron composition of *H. sapiens* compared to the other two species (see below). However, convergence between CDS/intron classification among the three species was reached at sequence size >600 bp with a classification rate >97%. By contrast, CDS/intron classification with CSF was higher for *D. melanogaster* and *H. sapiens* than for *A. thaliana* and was still <95% at 600 bp without a significant convergence trend (Fig. 1).

When considering CSF, we found that Sn and Sp vary in opposite ways across the sequence size range. This suggests the dependence of *τ_CSF_* from the sequence size. By contrast, both Sn and Sp of UFM increase with sequence size for all species, which indicates strong evidence of the independence of its threshold of both sequence size and species suggesting that it is a robust classifier (Table 1).

**Figure 2.**
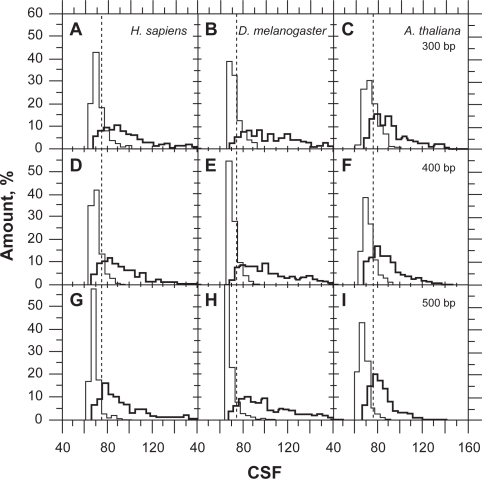
Classification of coding sequences (bold line) and introns (thin line) of *Homo sapiens* (A,D,G), *Drosophila melanogaster* (B,E,H) and *Arabidopsis thaliana* (C,F,I) at 300 (A,B,C), 400 (D,E,F) and 500 bp (G,H,I) by CSF. The dashed lines (CSF = 75) indicate the classification threshold (*τ_CSF_*). The sample size was 500 in both the introns and coding sequences.

**Figure 3.**
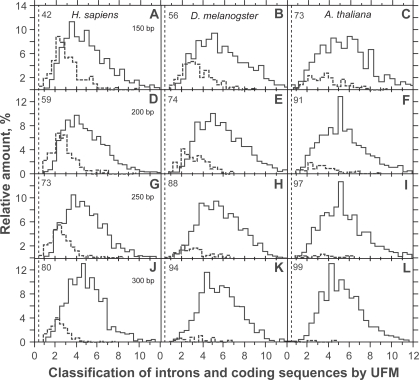
Classification of coding sequences (solid line) and introns (dashed line) of *Homo sapiens* (A,D,G,J), *Drosophila melanogaster* (B,E,H,K) and *Arabidopsis thaliana* (C,F,I,L) at 150 (A,B,C), 200 (D,E,F), 250 (G,H,I) and 300 bp (J,K,L) by UFM. The number on the upper left of each panel indicates the proportion of introns (%) that do not have any ORF with the purine bias of coding sequences for the size threshold considered. The sample size was 1000 in both the introns and coding sequences.

The CSF distribution has a strong right asymmetry with standard deviation and mean variance with sequence sizing (Fig. 2). This property is responsible for the variation of *τ_CSF_* with sequence size and cannot be solved by simple variable transformation such as log or square-root (not shown). By contrast, the asymmetry of UFM distribution is lower and false positives are due to intronic ORFs that have a purine bias compatible with that of CDSs (Fig. 3).

### Comparing the success rate of coding and non-coding ORF classification by UFM

Intermediary results of coding frame diagnosis by algorithm of UFM are summarized in Table 2 where we compared coding sequences (CDS) from *Homo sapiens* (Hs), *Drosophila melanogaster* (Dm) and *Arabidopsis thaliana* (At) (column 1, Sp) of variable sizes (column 2, Bp) and 1000 occurrences by sample (column 3, N). Data from this table show that *PBI* is always ≥1 in the coding frame (column 4, ΔF + ≥1). The number of CDSs whose putative coding ORFs are of the same size on “+” and “−” strands increases with the reduction of ORF size between 400 bp (~20%) and 150 bp (~70%) in *H. sapiens* and *D. melanogaster*. In *A. thaliana*, the probability of two ORFs having the same size is lower between 400 bp (~3%) and 150 bp (~40%) than in the other species (column 5, Bp+ = Bp−). The “−” strands of these sequences were all found to be *PBI* ≥ 1 (column 6, ΔF− ≥ 1), which is a potential source of false positives since *f_1_* ≥ *PBI*. Effectively, *f*_1_ is larger than 1 in almost all “+” strands of CDSs (column 7, F+1 ≥ 1). *f* can also be larger than 1 in the “−” strand (column 8, F−1 ≥ 1) and is found *f_max_* in the real frame −1 of CDSs, in the majority of these cases (column 9, Fr−1). *f_max_* can also occur in the real frames −2 (column 10, Fr−2) and −3 (column 11, Fr−3) of experimentally proven CDSs. Of course, these errors of coding frame diagnosis increase with the reduction of sequence size. However, we generally observed that *f*_+1_ > *f*_−1_ (column 12, Fr+1 ≥ Fr−1) and that the error rate occurring when *f*_+1_ < *f*_−_ (column 13, F+1 < F−1) is ≤ 5% for ORF > 250 bp (gray area). The error rate is generally the highest in frames −1 and −2 of experimentally proven CDSs and the frame where its maximum is found varies according to the species and sequence size (columns 14, Fr−1 and 15, Fr−2). The error rate in frame −3 remains marginal (column 16, Fr − 3).

Even if not sufficient, *PBI* ≥ 1 is a necessary condition for a sequence to be considered coding (step 4 of the algorithm, Fig. 3). We found that *PBI* values were scattered between 1 and 12 and were centered on 4–5 when UFM was run on CDSs (Fig. 3). Running the algorithm on introns led to disclose that values of most intronic ORFs were *PBI* < 1 (typically 0) and that a minority of ORFs were still *PBI* > 1 but <6, which means they have a purine bias similar to that of CDSs (false positives). The score of *PBI* associated with these false positives of coding ORFs was centered on 2 (Fig. 3). As pointed out above, the probability of an intronic ORF to be confounded with a CDS increases with the reduction of its size (Fig. 3). In *H. sapiens*, we found ~12% false positives when ORFs were 400 bp (Table 3). By contrast, the threshold of ~10% false positives occurred at about 250 and 200 bp in *D. melanogaster* and *A. thaliana*, respectively (Table 3).

The success rate of coding diagnosis in CDSs was close to 100% (Table 3). However, the rate of false positives in introns was too high with ~12% at 250 bp and 400 bp in *D. melanogaster* and *H. sapiens*, respectively. Consequently, a model for intronic ORFs is needed to improve this picture and should be inserted in step 4. In the absence of such a model, we tested the effect of filter 1, filters 1 + 2 and filters 2 + 3.

Filter 1 can be inserted in the UFM algorithm *a priori* as well as *a posteriori* because it does not come into conflict with the coding frame diagnosis. When filter 1 was inserted *a priori*, the rate of classification at 150 bp was found to be improved by only 1.6%, 0.7%, 0.1% in *H. sapiens*, *D. melanogaster* and *A. thaliana*, respectively. At 200 bp, the difference between *a priori* and *a posteriori* classification was <1% in the three species.

**Figure 4.**
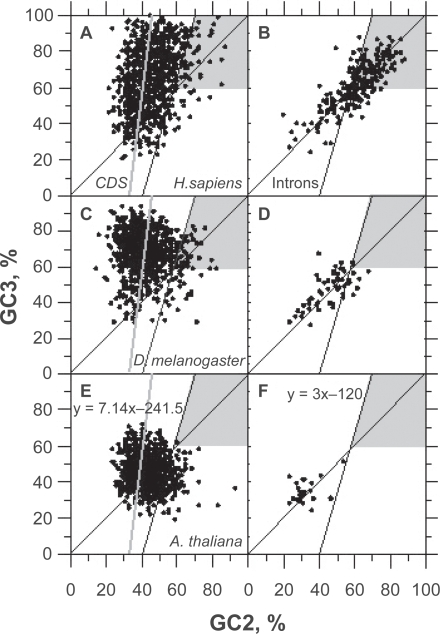
Plots of GC3 vs. GC2 in true positives (CDS, panels: A,C,E) and false positives (introns, panels: B,D,F) ORFs ≥ 250 bp after classification by UFM without filters. The sequence samples of *H. sapiens* (A,B), *D. melanogaster* (C,D) and *A. thaliana* (E,F) are the same as those used for Figure 3 and Table 3. The gray areas match ORFs corresponding to GC2 levels larger than the quantity (GC3 + 120)/3 when GC > 60% that are filtered out by filter 1. The gray line that matches y = 7.14 × 241.5 is for the *universal correlation*.^37^ The black line y = 3 × 120 matches the left border of the gray zone.

As shown in Figures 4A, C, E, the universal correlation is such that the GC2 level of coding ORFs is smaller than the quantity (GC3 + 120)/3 in 92% of the cases, provided that these ORFs are greater than 300 bp and with a GC level larger than 60%. This condition allows the elimination of about half of GC-rich intronic ORFs in *H. sapiens* (Fig. 4B) without significantly affecting the success rate of CDS diagnosis. Actually, non canonic true CDSs having a GC2 level larger than the quantity (GC3 + 120)/3 (Figs. 4A, C) only make up <3% of human CDSs larger than 200 bp (Table 3). Filter 1 does not affect false positive rates in *D. melanogaster* and *A. thaliana* because these two species do not carry intronic ORF whose GC2 level is larger than 60% (Figs. 4D, F).

By contrast to filter 1 and filter 2, filter 3 cannot be inserted in the UFM algorithm *a priori* because it is not only true in >94% of frames +1 of complete CDSs (data not shown), but also in ~60% of their frames −1 with the consequence that it would come into conflict with the success rate of coding frame diagnosis (data not shown). The addition of filter 3 to filter 1 (Table 3) was found to strongly decrease the false positive rate in *H. sapiens*. With this combination of filters, we reached the threshold of 5% false positives at <350, <300, <250 bp with a success rate of CDS diagnosis >90% in *H. sapiens*, *D. melanogaster*, *A. thaliana*, respectively (Table 3). However, *a posteriori* filtering with filter 3 could also be performed at step 5 of the algorithm. In that condition, we observed that it does not interfere with coding frame, however, it did not significantly improve the performance of the algorithmin comparison to the filtering by introducing filter 3 at step 6 (data not shown). This indicates that, in the case of filter 3, if an ORF candidate satisfies the condition G1 > G2, it will necessarily reach step 6.

The reasoning with filters 2 + 3 is, of course, identical to that with filters 1 + 3. The difference between success rates from both filter combinations is linear. The combination of filters 2 + 3 shows that it is possible to reach the threshold of 5% false positives at 250 bp even in *H. sapiens*, but it is at cost of the success rate of CDS diagnosis that comes down from >90% to ~80% (Table 3).

**Figure 5,:**
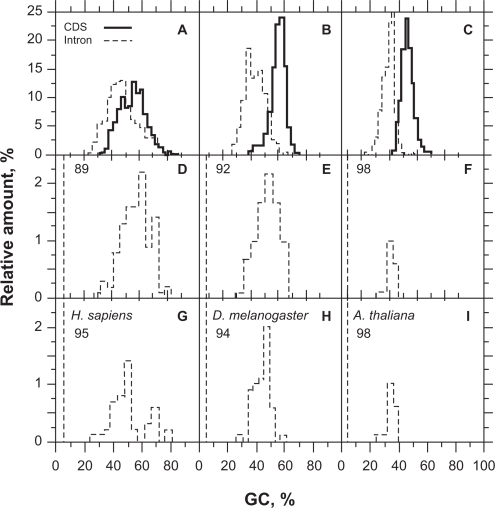
Compositional properties of CDSs (bold) and introns (dashed) in *H. sapiens* (A,D,G), *D. melanogaster* (B,E,H) and *A. thaliana* (C,F,I). Panels A,B,C show the relative amount of sequences (%) from Figure 3 and Table 3 classified by GC level (%). Panels D,E,F show the distribution of false positives (intronic ORFs classified as coding) resulting from ORF (≥250 bp) classification by filters 1 + 3. Panels G,H,I show the distribution of false positives (intronic ORFs classified as coding) resulting from ORF (≥250 bp) classification by filters 2 + 3. The numbers on the panels’ upper left indicate the proportion of intron sequences (%) that did not have any ORF with the purine bias of coding sequences for the size threshold considered.

The GC distribution of introns matches lower values than that of CDSs (Figs. 5A, B, C). In *D. melanogaster* and *A. thaliana*, GC is almost sufficient to classify coding and non-coding ORFs (Figs. 5B, C, respectively). In *H. sapiens*, the GC distribution of introns largely overlaps that of CDSs and such classification is more difficult to carry out as shown by the GC distributions of false positives in *H. sapiens*, *D. melanogaster* and *A. thaliana* (Figs. 5D, E, F, respectively). These distributions also show that false positives are especially difficult to filter out in the range of base composition between 40% and 60% GC (Figs. 5D, E). This is obvious when using filters 2 + 3 since the gain on false positive rate is at cost of success rate of CDS diagnosis (Table 3, Figs. 5G, H). Below 40% GC, the codon stop frequency is usually high enough to allow the elimination of ≥95% of non-coding ORFs ≥ 200 bp (Table 3, Figs. 4F, 5F, I).

**Figure 6.**
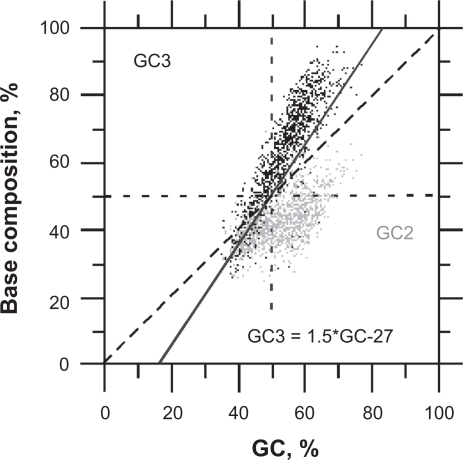
Relationship of GC2 (gray, *y* axis) and GC3 (black, *y* axis) vs. GC (*x* axis) in human CDSs (>600 bp). The solid line (GC3 = 1.5*GC-27) indicates the threshold of false positive filtering. This threshold has the same rate of false positive and true positive filtering as the threshold GC3 = 3*GC2–120 (Fig. 4). False positives of coding ORFs would stand on the diagonal of this plot (GC2≈GC3).

The difficulty of false positives filtering for GC level between 40% and 60% is due to the overlapping of GC2 and GC3 in CDSs, on the one hand, and in non-coding ORFs, on the other hand, in this interval of base composition (Fig. 6). This makes difficult to discriminate coding ORFs from pseudo-random sequences.

## Discussion

### General considerations on scoring the purine bias and stop codons

One specific feature that has been recognized to be general to all coding sequences (CDS) is the purine bias,^27^,^30^ i.e. the fact that the probability of finding a purine is higher in the 1st position of codons than in the 1st position of nucleotide triplets among any other five frames. The fact that the probability to find a purine is the highest in the 1st position of codons (*P_A_*_(1)_*P_G_*_(1)_) justifies the proportionality of a function *f* that maximizes this feature. By contrast, the product of probabilities of C in the 1st, G in the 2nd and A in the 3rd position of codons takes its minimum value in the coding frame of ~93% of complete CDSs,^30^ therefore, it is justified that *f* is inversely proportional to *P_C_*_(1)_*P_G_*_(2)_*P_A_*_(3)_ together with stop codon frequency (STOP), which is null in that frame. The absolute or relative frequency of stop codons can be used equally. However, the variation range of *f* is smaller using the absolute frequency (1 to 12) and that is why we used it in place of the relative frequency (1 to >80). Since the denominator cannot be equal to 0, and a constant is necessary; the best value for that constant is 0.01 (data not shown). Actually, this constant is a multiple of G2 whose range of variation is very limited over the whole biological spectrum (data not shown). The difference between the maximum of *f*, found in frame +1, and the minimum of *f*, found in another frame is expected to be higher in CDSs than in non-coding DNA since purine bias is not expected in random sequences. We can therefore use the information carried by the purine bias index (*PBI*) given by *PBI* = *f_max_* − *f_min_* to score the coding potential of a DNA sequence. This index is theoretically null in random sequences since *f* is the same in all frames with the consequence that *f_max_* = *f_min_*.

It is interesting to note here that the success rate of UFM classification is such that we can conclude that >80% of CDS > 250 bp follow the pattern of purine bias introduced by the ancestral codon.

### Comparing the success rate of CDS/intron classification by CSF and UFM

The sensitivity (Sn) measures the accuracy of classifiers in detecting coding sequences while the specificity (Sp) measures their accuracy in detecting introns. Therefore, the harmonic mean (F-score) of Sn and Sp is the best measure of the overall efficacy of a classifier in a coding/non-coding classification context. For this reason, we can conclude that UFM is at moment the best method of CDS/intron classification among the methods that do not need a training step (i.e. CSF, AMI and SRM).

The main advantage of UFM is that *f*_1_ is >1 in CDSs as short as 100 bp, which shows that the final decision by its algorithm concerning the coding status of an ORF can be improved with the inclusion of a better model for introns. This does not seem to be the case of CSF, AMI and SRM.

In the specific case of CSF a limitation of the method is due to the variation of the classification threshold (*τ_CSF_*)with the sequence size. This variation of *τ_CSF_* is responsible for the poor robustness of CSF as a classifier and makes it difficult to automate.

Given that CSF is scoring the codon asymmetry, the lower classification rate of *Arabidopsis thaliana* by CSF is probably due to the higher homogeneity in base composition of its CDSs compared to those of *Drosophila melanogaster* and *Homo sapiens*.

### Comparing the success rate of coding and non-coding ORF classification by UFM

Considering a CDS, it is obvious that the larger the ORF, the higher the probability that it matches the coding frame of a putative CDS. A corollary of this is that the higher the AT level of DNA, the stronger the statement. However, this consideration is true if the DNA under consideration is actually coding. If the DNA is not coding, the largest ORF does not make any sense. Therefore, the correct strategy is to search for the largest ORF among the potentially coding ORFs of both plus and minus strands. This means that a measure for the coding potential of an ORF is needed. A condition that could improve the success rate of CDS/intron classification should be better inserted in step 4 of the algorithm (*a priori*). However, to be effective it cannot come into conflict with the coding frame diagnosis of the putative coding ORF. In that case, it would decrease the success rate of the algorithm because it would generate alternative ORFs not corresponding to that of the coding frame of the actual CDSs (we understand by “actual CDSs” the ones proven through experimental investigations). That is what occurs when the condition G1 > G2 is inserted in step 4 of the algorithm. If this is done, the success rate of CDS/intron classification by UFM decreases because G1 > G2 is not only true in the coding frame of CDSs, but can also be true in other non-coding frames of these CDSs. An alternative to this process of *a priori* filtering is *a posteriori* filtering. The consequence of *a posteriori* filtering is that it does not maximize the coding ORF search; it just filters out false positives. A true coding ORF < 300 bp could be skipped by *a posteriori* filtering because it would not necessarily end up in the list of coding ORF candidates. This, together with statistical significance explains why true positives are increasingly lost when the size of ORFs under consideration is reduced (data not shown). *A posteriori* filtering is possible because a confident hypothesis of a coding frame is provided by *f* and allows testing the condition “G1 > G2” that only occur by chance in non-coding ORFs, at rather low frequencies.

*A priory* or/and *a posteriori* filtering allow acceptable success rates of CDS/intron classification in *H. sapiens* despite large heterogeneity of this genome.^38^ The higher rate of false positive elimination obtained through the use of these conditions shows that the ORFs on the diagonal of GC3 vs. GC2 are, indeed, quasi-random sequences.^39^ This is easy to establish for ORFs whose GC level is higher than 60%, but is difficult below this threshold. For that reason, we believe that filtering out false positives with these conditions is not very convenient and would be better replaced by a specific model for intronic ORFs. However, these simple filters can be useful for testing hypotheses since they allow coding ORF sampling according to objective and *universal* criteria.

False positives of coding ORFs of this study may have several sources. The most probable source of false positives may result from the activity of transposable elements. The cumulative intron invasion by transposable elements over time may be considerable and may carry coding sequences that may further evolve in pseudogenes.^40^

An alternative source of false positives could be lncRNAs.^41^ These RNAs (>200 bp) were shown to act in tissue specificity and to have regulatory functions, in particular on brain activity.^42^ They can be found in intergenic sequences as well as in introns. They were mostly described in vertebrates (human and mouse) and their most obvious origin is pseudogenes, which may justify the conservation of a purine bias in a quasi-random context.

## Figures and Tables

**Table 1.** Comparative analysis of CDS/intron classification by SCF and UFM.

**Species**	**Size, bp**	**CSF**			**UFM**		
		**Sn^1^**	**Sp^2^**	**F-score^3^**	**Sn^4^**	**Sp^5^**	**F-score**
*H. sapiens*	300	88.8	74.6	81.1	100.0	76.0	86.4
	400	86.6	87.6	87.1	100.0	88.0	93.6
	500	85.2	93.6	89.2	100.0	93.0	96.4
	600	84.2	93.4	88.6	100.0	97.4	98.7
*D. melanogaster*	300	95.2	71.4	81.6	99.8	97.4	98.6
	400	95.8	82.8	88.8	99.8	97.8	98.8
	500	93.4	89.6	91.5	100.0	98.6	99.3
	600	94.8	92.6	93.7	100.0	98.6	99.3
*A. thaliana*	300	90.8	59.6	72.0	100.0	100.0	100.0
	400	82.2	78.8	80.5	100.0	100.0	100.0
	500	78.6	90.8	84.3	100.0	100.0	100.0
	600	78.8	93.0	85.3	100.0	100.0	100.0

1 Sensitivity (%) of CSF for τ = 75.

2 Specificity (%) of CSF for τ = 75.

3 F-score (%) = 2*Sn*Sp/(Sn + Sp).

4 Sensitivity (%) of UFM for τ = 1.

5 Specificity (%) of UFM for τ = 1.

**Table 2.** UFM diagnosis of coding strand in CDSs with size (Bp) varying between 150 and 400 bp in the species (Sp) *H. sapiens* (Hs), *D. melanogaster* (Dm) and *A. thaliana* (At). The sample size (N) was 1000 throughout the experiment.

**Sp**	**Bp**	**N**	**ΔF + ≥1^1^**	**Bp + = Bp−2**	**ΔF** −≥ **1^3^**	**F + 1**≥**1^4^**	**F−1**≥**1^5^**	**Fr−1^6^**	**Fr−2**	**Fr−3**	**Fr+1** ≥ **Fr−1**	**F+1** < **F−1**	**Fr−1**	**Fr−2**	**Fr−3**
Hs	400	1000	1000	202	202	1000	178	122	44	12	981	19^7^	6	12	1
Dm		1000	1000	214	214	998	204	161	37	6	979	20	10	7	3
At		1000	1000	30	30	999	29	21	8	0	992	7	5	2	0
Hs	350	1000	1000	254	254	999	223	151	56	16	971	29	8	20	1
Dm		1000	1000	264	264	999	257	197	50	10	973	26	14	9	3
At		1000	1000	50	50	998	49	33	15	0	993	5	4	1	0
Hs	300	1000	1000	314	314	999	271	179	72	20	957	43	14	26	1
Dm		1000	1000	342	342	999	327	241	71	15	961	38	17	16	5
At		1000	1000	97	97	998	96	56	33	7	962	19	7	10	2
Hs	250	1000	1000	388	388	998	326	211	92	23	943	57	15	38	4
Dm		1000	1000	428	428	997	406	301	90	15	946	54	25	24	5
At		1000	1000	152	152	996	146	79	59	8	981	36	14	19	3
Hs	200	1000	1000	538	538	996	460	273	152	35	900	100	32	60	8
Dm		1000	1000	566	566	992	519	354	133	32	926	72	32	36	4
At		1000	1000	249	249	993	242	124	98	20	929	69	28	35	6
Hs	150	1000	1000	699	699	994	590	333	198	59	842	158	53	87	18
Dm		1000	1000	723	723	986	651	398	204	49	865	135	44	82	9
At		1000	1000	430	430	989	412	186	183	43	868	129	40	70	19

1 “ΔF+” is for *PBI* considering the “+” strand.

2 “Bp+ = Bp−” is to indicate that ORF size is the same for “+” and “−” strands.

3 “ΔF−” is for *PBI* considering the “−” strand.

4 “F+1” is for *f*_1_ considering the “+” strand.

5 “F−1” is for *f*_1_ considering the “−” strand.

6 “Fr−” is for the real frame of the CDS, i.e. Fr−1 for frame −1, Fr −2 for frame −2 and Fr −3 for frame −3, respectively,

7 The gray area is for error rate of coding frame diagnosis ≤5%.

**Table 3.** CDS/intron classification by UFM with filters 1, 2 and 3 in CDS and intron sequences of *H. sapiens, D. melanogaster* and *A. thaliana* varying between 150 and 400 bp.

**Species**	**Filters**	**Seq.**	**Size, bp**					
			**150**	**200**	**250**	**300**	**350**	**400**
*H. sapiens*	0	CDS*	100	100	100	100	100	100
		Intron**	57.7	40.6	27.5	20.5	15	11.7
	1	CDS	96.5	96.9	97.5	97.8	98.4	98.9
		Intron	47.0	29.8	17.1	10.5	7.0	4.2***
	1 + 3	CDS	85.3	87.9	89.2	92.6	93.5	94.5
		Intron	28.4	18.3	10.7	7.6	4.8	2.7
	2 + 3	CDS	78.1	81.0	82.6	85.0	86.5	87.8
		Intron	20.3	11.0	4.6	3.3	1.5	0.8
*D. melanogaster*	0	CDS	100	99.8	100	99.9	99.9	99.9
		Intron	43.6	25.8	12.2	5.6	3.7	2.6
	1	CDS	97.7	98.3	98.7	98.5	98.6	98.8
		Intron	43.6	25.8	12.2	5.6	3.7	2.6
	1 + 3	CDS	87.2	89.7	90.9	92.3	93.4	94.1
		Intron	25.6	13.2	8.4	3.9	2.3	1.8
	2 + 3	CDS	81.3	82.2	83.6	86.0	87.4	88.5
		Intron	21.5	11.5	5.8	3.1	1.8	1.4
*A. thaliana*	0	CDS	99.7	99.8	99.9	100	99.8	99.9
		Intron	27.1	9.1	2.6	0.8	0	0
	1	CDS	99.7	99.7	99.8	100	99.8	99.9
		Intron	27.1	9.1	2.6	0.8	0	0
	1 + 3	CDS	86.9	88.3	90.9	92.7	94	94.4
		Intron	16.2	5.3	1.8	0.7	0	0
	2 + 3	CDS	76.2	77.0	79.3	83.2	85.3	85.9
		Intron	16.2	5.3	1.8	0.7	0	0

* “CDS” indicates the proportion (%) of CDS that were correctly classified by the corresponding algorithm, i.e. the true positives. The CDS that are not detected, i.e. the false negatives are missing from the CDS output list.

** “Intron” indicates the proportion (%) of introns that were wrongly classified, i.e. the false positives. The non-coding sequences correctly classified do not appear in the output list. All entries whose values is >0 contain an ORF whose purine bias is typical of a CDS for the size threshold considered.

*** Gray areas indicate cases where the false positive rate of coding ORF diagnosis is below or close to 5%.
